# The effects of an online student question-generation strategy on elementary school student English learning

**DOI:** 10.1186/s41039-015-0023-z

**Published:** 2015-11-19

**Authors:** Fu-Yun Yu, Yu-Ling Chang, Hui-Ling Wu

**Affiliations:** 1 Institute of Education, National Cheng Kung University, No. 1, University Rd, Tainan City, 701 Taiwan; 2 Hsin-Chung Elementary School, No. 30, Hsing-jhong, Chia-Yi County Taiwan

**Keywords:** Computer-assisted learning, Drill-and-practice, English as a second language, Language education, Student question-generation

## Abstract

Recognizing the potential of online student question-generation to engage language learners in communicative activities and use the target language in a personally meaningful way for language and learning motivation development, an experimental study examining the English learning effects of this approach, in comparison to an online drill-and-practice strategy, was conducted. A quasi-experimental research design was adopted. Four sixth-grade classes (*N* = 106) participated in this study and were randomly assigned to different treatment groups. An online learning system supporting the various learning activities was adopted. The results of analysis of covariance (ANCOVAs) showed that students in the online student question-generation group performed significantly better in English assessments and exhibited higher learning motivation than those in the contrast group. The significance of this study and suggestions for instructional implementation and future works are also presented.

## Introduction

### The importance of English language learning in a global society of lifelong learners

English has become one of the most popular languages in the world and is recognized as the most widespread language for communication in education, technology, business, diplomacy, science, and sports, as well as the high-tech and service-oriented industries, and many other fields (Zhang 2011). According to a report published by the British Council (2013), English is spoken at a useful level by some 1.75 billion people worldwide (i.e., one in every four people). Moreover, it is estimated that English has official or special status in at least 75 countries, that more than two thirds of the world’s scientists and researchers use English as a second language, that 80 % of the world’s electronically stored information is in English (Davies and Patsko 2013), and that two billion people will be using it or learning to use it by 2020 (British Council 2013).

The importance of encouraging people to learn English is widely acknowledged throughout Asia, in order to improve both national and individual competitiveness in an increasingly globalized world. Moreover, English has gradually become either the official or dominant foreign language in many Asian countries (Feng 2011). For example, in the 1980s, only approximately 4~5 % of Indians spoke English (Crystal 2004), but in recent years, it has been estimated that more than one third of the population of India (i.e., 350 million people) are able to use English in conversation (Graddol 2010). Overall, roughly 22.8 % of the population in Asia is able to speak English (Bolton 2008).

In light of the increasingly high demand for English proficiency, in 2001, the Ministry of Education in Taiwan launched the Nine-Year Joint Curricula Plan to revise the Elementary and Junior High School education system (Chern 2002). One of the major changes was that English became a required course for all fifth grade students and onwards, as opposed to such classes only starting in junior high schools (Wang 2008). Two years later, in 2003, the age at which formal English language education began for Taiwanese elementary school children was further lowered to the third grade (Chou 2008).

The efforts to promote English proficiency in Taiwan were also expanded to tertiary education and in-service professional development. For instance, starting from 2002, the Ministry of Education encouraged universities to develop regulations for students to pass a certain level of standardized English proficiency tests as part of their graduation criteria (Hsu 2009; Wu & Wu 2010). Some government employees are also required to demonstrate a minimum A2 level in the Common European Framework of Reference for Languages (CEFR) (Wu & Wu 2010).

In spite of these reforms, Taiwanese students’ performances in international English standardized tests still fall behind those of comparable Asian countries. According to a report by Educational Testing Services, the TOEFL scores of Taiwanese test-takers have been the lowest among those from Mainland China, Korea, Hong Kong, and Singapore since 2004 (Cheng 2011; Educational Testing Services 2010). As such, devising ways to improve English language teaching with substantiated learning effects remains a key concern for English scholars, educators, and government officials in Taiwan.

### Teaching approaches to language education: from behavioristic to communicative paradigm shifts

According to Warschauer and Meskill (2000), language instruction has shifted from behavioristic to communicative paradigms over the last few decades. For many years before this, language instructors mainly focused on behavioristic instruction, using methods such as grammar translation and audio-lingual approaches, which utilize repetitious drills and practices in order to help students to memorize the focal language. In grammar translation lessons, teachers present students with large amounts of vocabulary and grammatical rules (Wang 2009), with the idea being that memorizing these can aid in the translation of sentences and longer texts (Kong 2011). To this end, students often engage in the tedious memorization of seemingly endless lists of grammar rules and vocabulary items (Liu & Shi 2007). Similarly, instructors who adopt audio-lingual methods to teach a second language tend to demonstrate correct models of sentences first, and students then repeat the accurate pronunciation and intonation of the target language verbally in order to memorize these (Felder & Henriques 1995; Wang 2009). These traditional teaching methods, in accordance with behaviorism, emphasize that instruction is composed of creating “a process of mechanical habit formation” by providing many opportunities for students to practice (Richards 2005).

However, over the last decade, a growing number of scholars have opposed the extensive use of these traditional language teaching strategies, with their focus on drills and practices. In their opinion, these approaches are mainly aimed at helping students to pass reading and writing examinations, rather than being able to communicate naturally in real-world contexts (Butler 2005; Su 2006; Yang & Lyster 2010). Moreover, traditional language teaching methods also prevent students from taking responsibility for their own learning (Ellis 2008).

In light of these weaknesses, many scholars have proposed alternative language instruction methods, such as total physical response, the natural approach, silent way, or suggestopedia, which have been applied since the 1970s (Brandl 2007). These approaches avoid rote learning, and instead focus more on the learners and their cognitive processes when learning a language, with learning being seen as a social process for conveying meaning (Jacobs & Farrell 2003; Nunan 2004). In simple terms, many scholars now see the ultimate goal of learning a language as being able to communicate effectively (Brandl 2007). As a result, a growing number of researchers now recommend that educators use communicative approaches, which encourage students to apply the target language to create meanings in various contexts (Wang 2009).

The emphasis on communicative approaches to language instruction (i.e., communicative language teaching, CLT), as both the means and ultimate goal of language learning, fits well with the principles of constructionism (Ruschoff & Ritter 2001). It is suggested that learners exposed to CLT (e.g., task-based instruction, output-based production tasks) are more inclined to engage in meaningful externalization and internalization of their own linguistic resources, rather than attempting to memorize the accurate models of the language (Richards 2005; Swain 1993). The process of using language to realize one’s communicative intent helps learners to pay attention to both the accuracy of forms and the meanings of language simultaneously (Izumi & Bigelow 2000), engage in deeper and more elaborate processing of the forms (Izumi 2002), and seek solutions to any communicative problems encountered autonomously, once any gaps between their linguistic knowledge and intended goals are noticed (Hanaoka 2007; Swain & Lapkin 1995). As a result, CLT enables learners to increase their linguistic awareness and build their capabilities through actively and continuously using the language to communicate in meaningful contexts or around purposefully designed tasks (Richards 2005; Swain 1993).

In sum, CLT emphasizes learner-centeredness in language education (Song 2009). Also highlighted are the notions of providing learning opportunities for active engagement and some control of the learning process to facilitate greater self-competency, which in turn results in greater personal meaning and affective gains (Chang & Ho 2009). Consonant with the ideas of CLT, the current study applies a student question-generation strategy to language instruction, and its effects on language performance and learning motivation in English are examined.

### Student question-generation: empirical evidence supporting its educational value and as an alternative approach to CLT for English learning

Research that applies the student question-generation (SQG) strategy in various fields has generally found positive effects with regard to outcomes such as comprehension (Brown & Walter 2005; Drake & Barlow 2008), motivation (Chin et al. 2002), positive attitudes toward the subject matter studied (Keil 1965; Perez 1985), problem-solving abilities (Dori & Herscovitz 1999), cognitive and metacognitive strategy development (Yu & Liu 2008), intra-group communication (Yu & Liu 2005), and more diverse and flexible thinking (Andre & Anderson 1978-79; Brown & Walter 2005; English 1997). While there is a growing body of empirical evidence substantiating the educational value of SQG, a majority of related studies deal with math and natural science subjects, and there are still a limited number of works exploring its effects in language instruction (Yu & Lai 2014).

As noted above, and consistent with the notions of CLT, SQG may prompt students to use English for meaningful oral and written communication. By requiring students to generate questions and corresponding answers based on the English learning content, as if they were test item designers, it is assumed that SQG can help students to develop language proficiency by consciously directing their attention to the forms and meanings of the target language simultaneously during SQG activities. To elaborate, when students engage in question-generation, the opportunities this provides to record questions by themselves may direct their attention to the correct pronunciation of the target language. To ensure that their speech is audible and clear, it is likely that students will engage in numerous rehearsals before recording, as well as listening to their recordings to make sure that they are of acceptable quality. In addition, in order to provide the correct answers to the questions they compose, students are likely to review the related learning materials so that they can better understand the content. This process may further direct students to review their questions and engage in editing and re-writing, as needed. In short, instead of attempting to memorize the target language, as seen in more traditional approaches, SQG can enhance the integrated development of listening, speaking, reading, and writing proficiency in language instruction, with its focus on using the target language for real communication, rather than simply passing tests.

### Research purposes and questions

This study examines the effects of SQG and traditional drill-and-practice strategies on English performance. In order to encourage the students to engage in all four skills of listening, speaking, reading, and writing (and thus not only the latter two), this work carried out SQG in a computer-supported context, with online SQG chosen as the focal task. Furthermore, since learning motivation is very important for second language learners (Dörnyei & Ushioda 2011; Murray et al. 2011), and the communicative use of language has been suggested to increase this (Sanchez 2004), this work also examines the effects of online SQG on the student learning motivation.

In short, this study examines the effects of online SQG on English learning in comparison to the online drill-and-practice (D&P) strategy, guided by the following two research questions:

1. Are there any significant differences between the online SQG and D&P strategies with regard to their effects on student academic performance in English?
2. Are there any significant differences between the online SQG and D&P strategies with regard to their effects on student learning motivation in English?

## Methods

### Participants and instructional materials

Four classes of sixth-grade students (*N* = 106; 46 male, 60 female) taught by the same instructor from one primary school in Taiwan participated in this study. Because the participating students had started taking computer classes since they were in the third grade (one 40-min instructional session per week), they possessed the skills needed to participate in the online learning activities designed in this study, including word processing, imaging editing, multimedia file uploading, and web surfing. Additionally, since the students had been taking English classes since the third grade (two 40-min instructional sessions per week), they possessed the basic English abilities needed to engage in the focal activities. More specifically, all the participants had already learned the English alphabet and phonics, as well as the vocabulary and basic sentence structures frequently encountered in daily life.

The instructional materials adopted by the participating school, volume 7 of *Hi English* (Hess International Educational Group 2008), were used in this study. The vocabulary, sentence patterns, and phonics covered in the three units used in this work are listed in Table 1. Students should be able to use these sentence patterns to ask and answer questions in verbal and written forms after receiving the instruction. They should also be capable of differentiating and naming the various phonics in both verbal and written forms. Finally, the students should be able to use the taught vocabulary to describe their feelings or certain objects.

**Table 1:** Main topics covered in the English class during the study

Unit number/title	Vocabulary	Sentence Pattern	Phonics
1: Do you see any lions?	Lion, elephant, tiger, pig, snake	How many… do you see?	Sm—small, smart Sn—snack, snake Sw—sweater, swing
		Do you see any …? Yes, I do. No, I don’t.	
2: Look! There’s an elephant.	Big, small, clean, dirty, long, short	It looks … They look…	Sk—skirt, sky Sp—spoon, spider St—star, stairs
		There is a … There are …	
3: I feel tired.	Excited, tired, sick, bored, sleepy	How do you feel? I feel…	
		How do you feel? I feel … I want to …	

### Online learning system

An online learning system called QuARKS (Question-Authoring and Reasoning Knowledge System) (Yu 2009) was adopted in this study. Like all similar online systems, multimedia contents, including pictures, audio, and video, can be embedded as part of the questions that the students generate or answer. Texts with different fonts, styles, colors, and sizes can also be used for differentiation or highlighting purposes. Moreover, the question types that are often employed at all levels of the educational system are supported in QuARKS. Nevertheless, since multiple-choice, true-or-false, and matching questions are most frequently used by elementary English teachers in Taiwan, these were chosen to be generated for the online SQG and practiced in the D&P activities, which are described in more detail below.

**Fig. 1:**
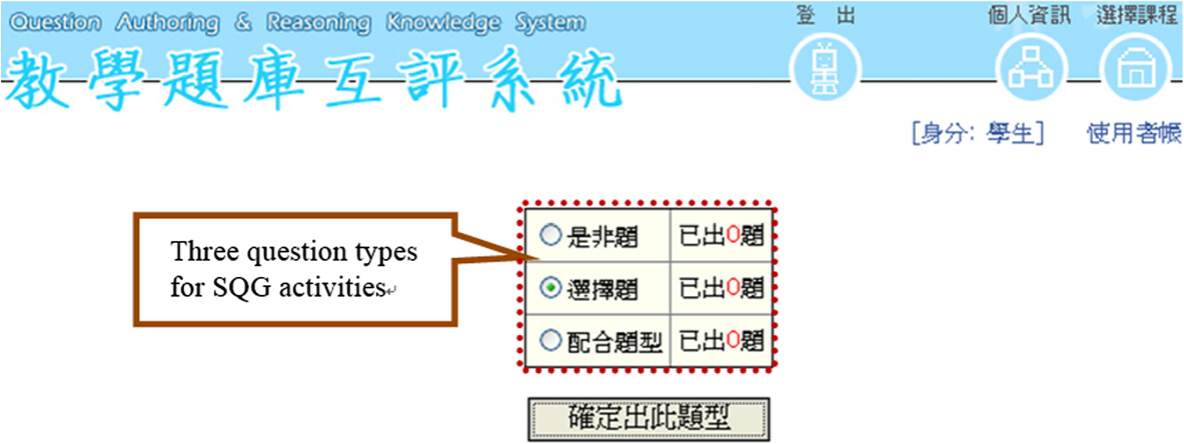
Selection of a question type to generate in the online SQG system

**Fig. 2:**
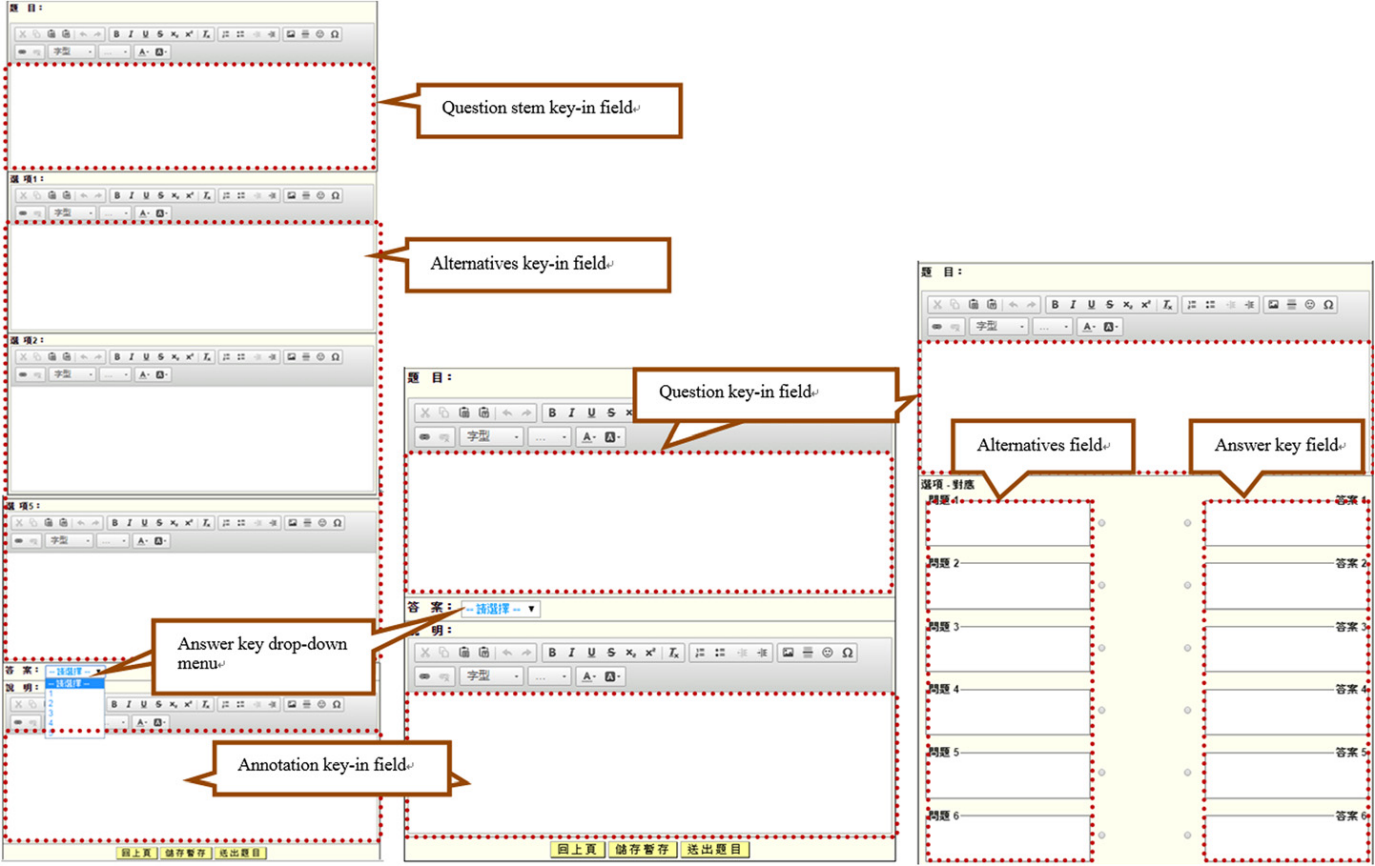
Online SQG space: multiple-choice (*left*), yes/no (*middle*), and matching (*right*) questions

**Fig. 3:**
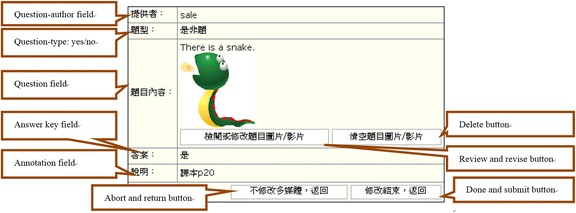
Retrieval of questions generated for review, revision and deletion

For the online SQG, after selecting the type of questions to generate (by clicking on the respective icons, Fig. 1), the students were directed to the related space for the question-generation activities (Fig. 2). The students then needed to enter information into all the required fields for a successful submission. Specifically, for multiple-choice questions, the students needed to provide a question stem, two to five alternative answers, an answer key, and an annotation briefly explaining the main ideas tested in the question for each item (see Fig. 2). For yes/no questions, the students needed to provide a question, an answer key (yes or no), and an annotation for each item. Similarly, for matching questions, the students needed to give a question, two to ten options, and an answer key for each of the options to be matched. Once questions are submitted, they can be retrieved, revised, and deleted by the question-author (see Fig. 3).

**Fig. 4:**
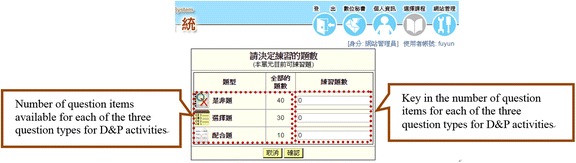
Specification of the number of questions in the online D&P window

**Fig. 5:**
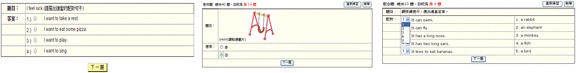
Online D&P activities: multiple-choice (*left*), yes/no (*middle*), and matching (*right*) questions

**Fig. 6:**
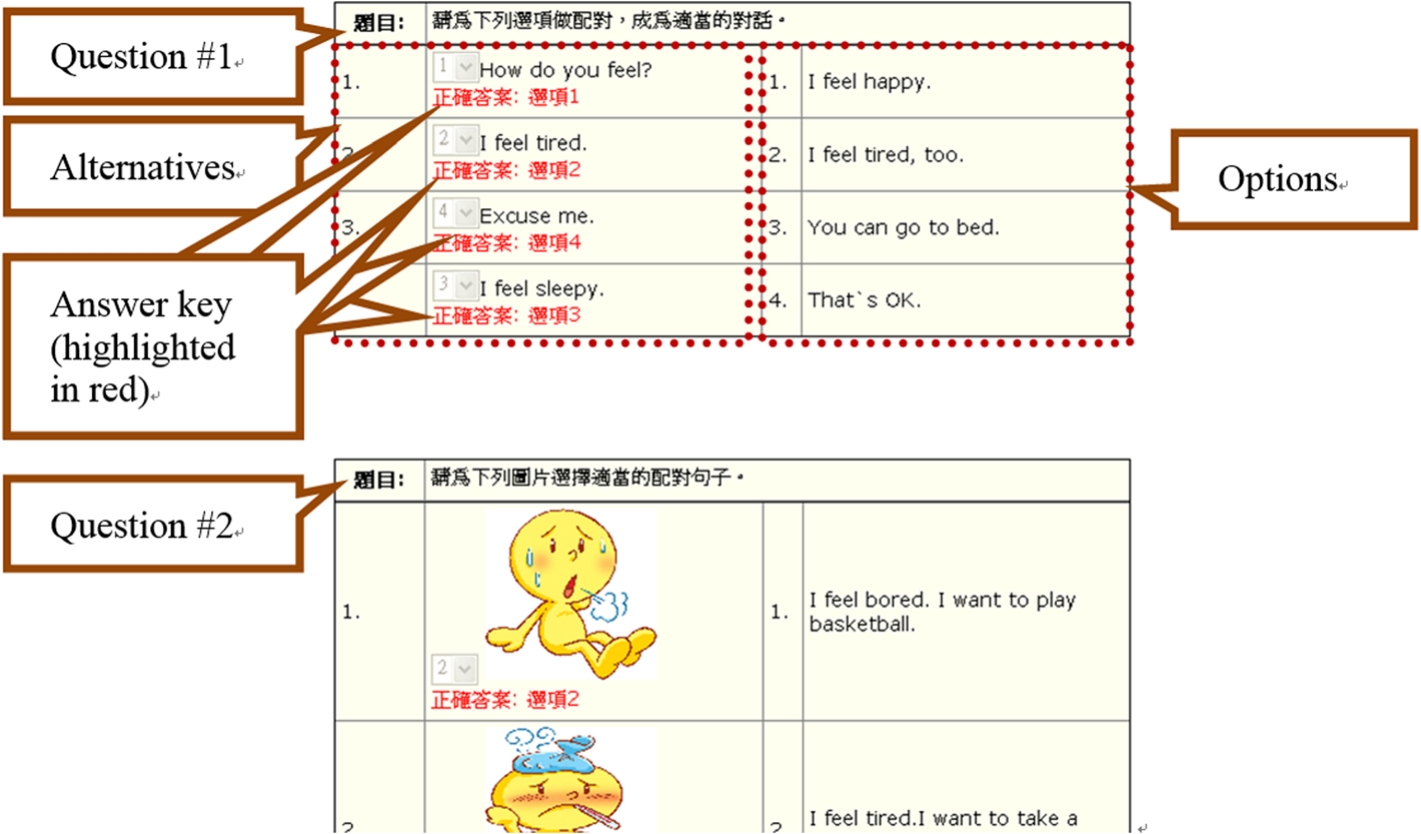
Online D&P review with correct answer shown (matching question type)

For online D&P, the students first keyed in the number of items they would like to answer in the boxes beside the respective different question types (see Fig. 4) and were then directed to the related D&P spaces (see Fig. 5). After completion, the students could review all the questions they had answered during the D&P session, along with the related answer keys (see Fig. 6). If there was enough time, the students could then choose to do the drill-and-practice activity again by re-entering the number of questions to answer. The system would then randomly re-select the specified number of questions from the online database and put them in a different order, with the various options for multiple-choice items also re-sequenced.

### Research design and the experimental treatment groups

This study adopted a pretest-posttest quasi-experimental research design that lasted for 11 weeks. Each of the four intact classes used the adopted system for participation and was randomly assigned to one of the two treatment conditions: online SQG (the experimental group, *N* = 54) and online D&P (the contrast group, *N* = 52).

The students assigned to the online SQG group were directed to compose questions with answers in their assigned groups in accordance with that week’s instructional content in text or multimedia forms. Students could choose to select the files already stored by the instructor in the multimedia database, or upload any files they found on the internet or created themselves (e.g., photos taken with their smartphones or digital cameras). They could also record audio of themselves asking questions.

In contrast, students in the online D&P group engaged in rote learning activities by answering a series of questions similar to those in the traditional English practice sessions with their assigned groups on computers. All questions were purposively selected by the instructor from the item bank provided by the textbook publisher.

### Experimental procedures

To ensure the appropriateness of the implementation procedures, the smoothness of the adopted online system, and the clarity of the instrument, two pilot tests were conducted prior to the actual study in the previous semester. The main aims of the first pilot test were to ensure that the instruction and performance criteria for question-generation for different question types were understandable and clear to the students with different levels of English proficiency. Six sixth-grade students with below-average, average, and above-average English proficiency were thus purposefully selected and invited to participate in this pilot test. Some adjustments were made based on the participants’ feedback with regard to the activity (i.e., more question models given to illustrate the performance criteria of SQG).

Furthermore, to ensure the operational smoothness of the adopted system in a large class setting, the appropriateness of the time allocated for different instructional events (i.e., training, hands-on practice with the system, and feedback on student performance in the last session), and the comprehensibility of the motivation scale, a second pilot test was conducted. Two fifth-grade classes (*N* = 59) participated in this, one for the online SQG and the other for the online D&P. The results of the pilot study confirmed the system’s stability and instrument’s clarity. Also, based on data collected during the second pilot study, the maximum numbers of items included for different question types for each online D&P activity were as follows: 30 items for multiple-choice questions, 40 for true/false, and ten for matching.

Afterwards, the actual study was conducted in the students’ regular English instructional sessions in the English classroom. In view of the value of collaborative learning for knowledge creation (Paavola & Hakkarainen 2005), the participants were assigned to groups of three or four to work collaboratively during the study. In accordance with the suggestions of most CL researchers (e.g., Johnson & Johnson 2009), heterogeneous groups, based on the students’ English grade in the previous semester were formed. Specifically, for groups of three, there was one student from the lower quartile, one from the upper quartile, and one from the interquartile range. In constrast, for groups of four, there was one from the lower quartile, one from the upper quartile, and two from the interquartile range. As a result, eight groups were formed in each participating class.

**Fig. 7:**
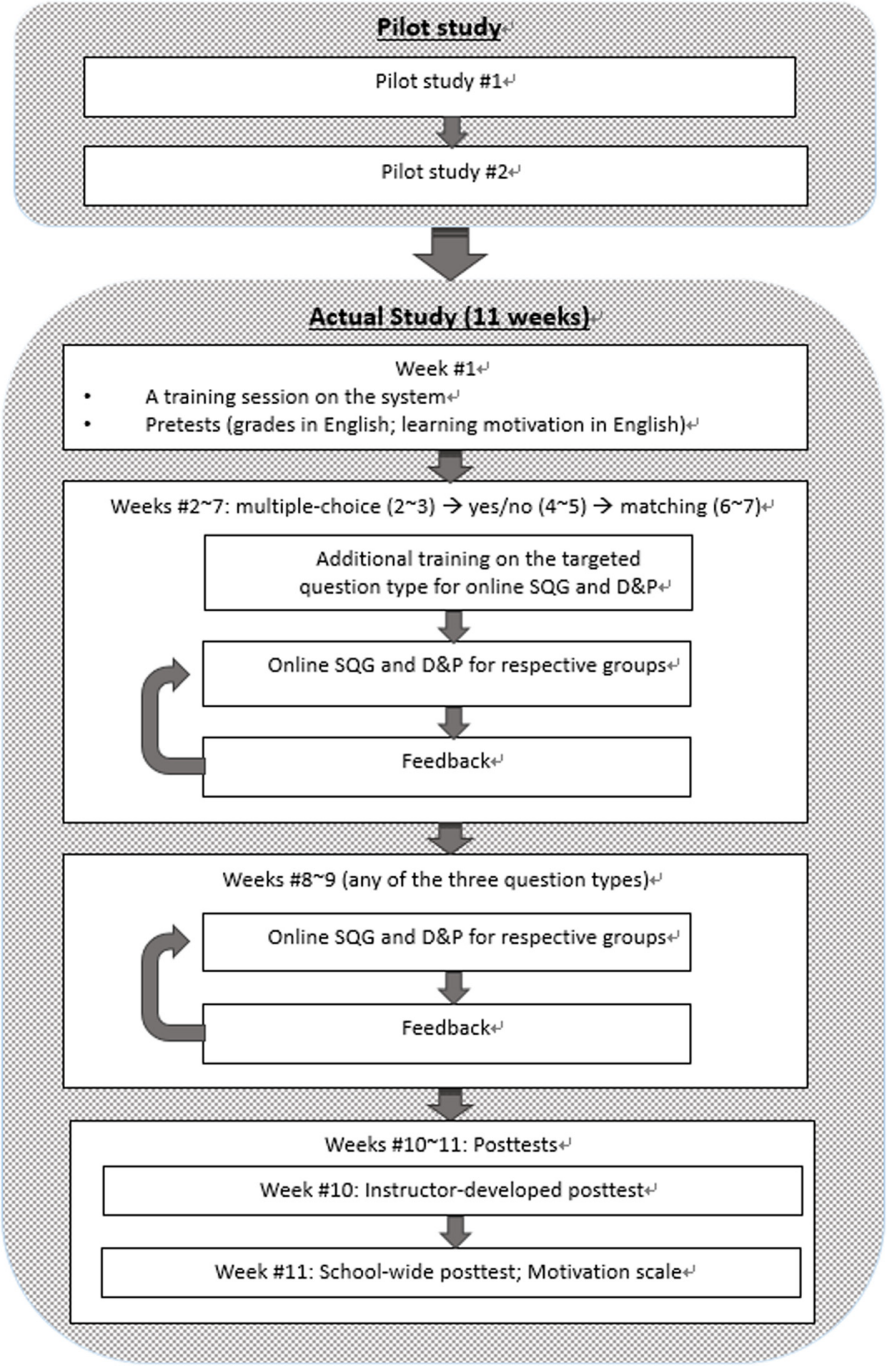
Experimental procedures used in this study

As a routine during the study, the students participated in the online learning activities in the last 20 min of the second instructional session each week for 11 weeks. In the first week, a training session was given by the instructor to help the students get acquainted with the general operational procedures of the system, and they then completed the motivation scale, and their English grades from the previous semester were collected. One of the three question types was then targeted every two weeks (i.e., weeks 2 to 3 for multiple-choice, weeks 4 to 5 for yes/no, and weeks 6 to 7 for matching). To ensure the students had the necessary skills, a brief training session on generating or answering the target question type on the adopted system was arranged before the students took part in the related online activities (i.e., online SQG for the experimental group and online D&P for the contrast group). Feedback on student performance was arranged the following week, in which exemplary student work was highlighted and discussed. After gaining experience in generating or answering each of the three question types using the system, in weeks 8 and 9, the students were allowed to choose their preferred question types to generate or answer during the activities. In total, the students engaged in eight sessions of online SQG or online D&P activities in their respective groups. The instructor-developed posttest was then administered in the tenth week, while in the 11th week, all the participants took the school-wide posttest and completed the motivation scale. A flow diagram summarizing the major experimental procedures used in this study is shown in Fig. 7.

### Instruments

#### English academic achievement tests

The students took instructor-developed and school-wide administered academic achievement tests after participating in eight online learning activities to measure their English performance. The instructor-developed test was composed of 28 multiple-choice, ten true/false, and three matching questions (with 12 options/answers). The school-wide administered test was composed of ten multiple-choice, eight true/false, and four matching questions (with 32 options/answers). Multimedia files were included to assess student recognition and use of the learned English (and thus not merely their memorization). Audio recordings were also included to assess student listening comprehension.

All items underwent item analyses procedures and needed to meet the criteria set for item discrimination (.25) and item difficulty (.40) before they were included for data analysis. For the instructor-developed test, all the items except one yes/no question met the set criteria, and thus only one item was excluded from the data analysis. For the school-wide administered test, since all test items met the set criteria, all were included in the data analysis. Finally, the Cronbach’s α for the instructor-developed and school-wide tests were 0.93 and 0.98, respectively, indicating their high reliability.

#### Learning motivation in English

Due to the context sensitive nature of motivation (Duncan & McKeachie 2005), Pintrich, Smith, and McKeachie’s Motivated Strategies for Learning Questionnaire (MSLQ) was modified by Shiu (2002) to assess student learning motivation in English, and was adopted in this study. The scale contains 35 items which assess the respondent’s learning goal orientation, task value, control beliefs, self-efficacy, expectations of success, and test anxiety in English. For each item, the students rated themselves on a five-point Likert scale, from strongly disagree (1) to strongly agree (5). After the scores of negative items were reversed (i.e., items on test anxiety), they were added to the scores of all other items to represent the respondent’s learning motivation in English in the data analysis. The higher the score was, the greater the learning motivation the students had with regard to English.

After passing the expert content validity procedure, exploratory factor analysis was conducted. With all items having at least a factor loading of 0.45 on the corresponding factor, and all constructs accounting for a total of 81.6 % of the variance, the scale thus had satisfactory validity. Finally, the Cronbach’s α of .92 indicated the consistency of this scale.

## Results

### English academic achievement

Tests of analysis of covariance (ANCOVA) were conducted to examine the comparative effects of the online SQG and D&P strategies on student academic achievement in English, with student grades in English from the previous semester as the covariate. The assumption of the homogeneity of regression slopes was satisfied for both the instructor-developed test, *F*(1, 102), 1.714, *p* > .05, and school-wide administered test, *F*(1, 102) = 1.527, *p* > .05, before proceeding to ANCOVA. The results of ANCOVA indicated that there were significant differences in English performance between the two groups, *F*(1, 103) = 5.461, *p* < .05, *F*(1, 103) = 5.004, *p* < .05, for the instructor-developed and school-wide administered tests, respectively, after controlling for student English performance in the prior semester. As shown in Table 2, students in the online SQG group had a significantly higher adjusted mean test score in both the instructor-developed and school-wide administered tests than those in the online D&P group.

**Table 2:** Descriptive statistics of the experimental treatment groups for the observed variables

Observed variables	Two treatment groups	Online SQG (*n* = 54)	Online D&P (*n* = 52)
English academic achievement	Pretest^a^		
	Mean (SD)	76.37 (21.13)	79.09 (20.25)
	Posttest (instructor-developed)		
	Mean (SD)	78.71 (20.66)	75.81 (22.73)
	Adjusted Mean	79.94	74.53
	Posttest (school-wide)		
	Mean (SD)	82.61 (21.29)	73.19 (27.26)
	Adjusted Mean	84.63	71.86
English learning motivation	Pretest^b^		
	Mean (SD)	123.15 (20.77)	111.06 (21.83)
	Posttest		
	Mean (SD)	126.29 (19.18)	110.00 (21.93)
	Adjusted Mean	122.13	114.02

^a^Grades in English from the previous semester

^b^Score on the modified MSLQ

### Learning motivation in English

ANCOVA was also conducted to compare the learning motivation effects of online SQG and D&P, with the student pretest scores on the modified MSLQ as the covariate. The homogeneity of regression tests showed no violation of the assumption, *F*(1, 102) = 1.805, *p* > .05. The results of ANCOVA revealed that there were significant differences between the two groups in learning motivation after the influence of the pretest was controlled, *F*(1, 103) = 7.326, *p* < .05. As shown in Table 2, students in the online SQG group scored higher in the modified MSLQ (adjusted mean = 122.13) than those in the online D&P group (adjusted mean = 114.02).

## Discussion

### Online SQG promoted English performance to a greater extent than online D&P

The findings of this study indicated that online SQG was a more effective strategy to enhance student English performance than the online D&P approach. In order to come up with test-worthy ideas and present them in an appropriate form of questions (i.e., text-only or text with audio and pictures), students who engaged in the SQG activities would be more likely to put the learned language into actual use and practice listening, speaking, reading, and writing with it, as compared to those who completed the online D&P exercises. In fact, there was little if any need and few opportunities for the D&P group to practice speaking English. Moreover, different ways of connecting and combining the vocabulary items and sentence patterns learned from the current unit, as well as different units, were more likely to be tried during the SQG process.

As seen in the questions generated by students, many items contained audio recordings or photos produced by the participants themselves and used to describe the abstract concepts covered in the units (e.g., excited, tired, sick, bored, sleepy, big, small, clean, dirty, long, and short). Some even included vocabulary and sentence structures covered in prior units. Such an elaboration process, according to King (1995), helps to build a complex cognitive network and reflects what McCrindle and Christensen (1995) emphasized as a powerful and effective way to enhance meaningful learning. In this regard, similar to what CLT can offer, the SQG activities would direct students to pay more and closer attention to the forms and meanings of the language, rather than merely fixating on the correctness of grammar, spelling, and pronunciation, as seen in the more behaviorist approaches to language teaching (e.g., grammar translation and audio-lingual method). The results of this study also supported the output hypothesis, as the process of output production allowed students to be more conscious of their linguistic states (Swain 2005).

In contrast, the online D&P activities had weaker effects on English performance. The teacher’s informal in-class observations revealed that students in the D&P group mainly engaged in repeated practice. When encountering problems, these students were less often seen to adopt cognitive or metacognitive strategies, such as searching for resources, discussing things with peers, or asking the teacher questions, than those in the SQG group. This anecdotal observation is in consonance with the finding of McLoughlin and Oliver (1998) that students rarely use high-level cognitive processes in D&P activities. Instead, they tend to focus on practicing tasks in a repetitive manner until mastery is achieved.

In sum, the different levels of cognitive processing involved in SQG and D&P may result in the different learning effects obtained in this study. The various cognitive processes mobilized and enacted by SQG (e.g., reflecting on the learning experience, making sense of the learning materials, searching one’s existing knowledge reservoir for possible connections and appropriate use of the language, seeking additional information for possible alternative answers and options for questions, connecting newly learned ideas with prior knowledge) were found in this study to enhance student English performance in different language areas (i.e., reading, writing, and listening), to a greater extent than was seen with D&P.

### Online SQG enhanced learning motivation to a greater degree than online D&P

Students’ responses to the learning motivation scale indicated that those assigned to the online SQG group, as compared to those to the online D&P, were more motivated to learn English, and tended to agree more with statements such as: learning the content covered in English class is very important to me; it is my own fault if I do not learn the materials presented in English class; and I am confident that I can understand the most complex material delivered by the instructor in this course. In other words, through the experience of constructing rather than simply answering questions online, students were generally more appreciative of the task value of the focal activity, were more intrinsically than extrinsically motivated, were more positive about their performance in English, were less anxious about English tests, and felt more that they themselves should be responsible of their own learning.

Although D&P strategies have traditionally dominated language learning activities, over the last few decades, the focus has shifted from a teacher-oriented to learner-centered pedagogy (Hoven 1999). Instead of using repetitious D&P to strengthen student memorization of the target language, a learner-centered pedagogy stresses the importance of encouraging learners to communicate in a meaningful context. The online SQG strategy was thus proposed to better meet the needs of the communicative approach. In this, students are given reasons to communicate using the target language and opportunities to produce something concrete in different forms (such as text-based or multimedia items) during the SQG process. As a consequence, the online SQG strategy was found to be more effective in promoting student learning motivation than the online D&P approach. This result is consistent with the suggestion of Sanchez (2004) that communicative use of the target language can increase learning motivation.

## Conclusion

Since second language learners do not fully understand the language they are learning, and often do not need to actively use it for daily communicative purposes, as would be the case when learning a first language, the learning process tends to be less effective, and students have less motivation to engage in learning (Masgoret & Gardner 2003). It is thus necessary to cultivate an environment that can encourage the communicative use of the second language to increase both learner motivation and language competencies. This is even more urgent for students in East Asian countries where there are few opportunities for speaking and writing English in daily life, and the learning experiences of students in such contexts are often rather negative (Hwang et al. 2010).

In an attempt to cope with these limitations, this study proposed an online SQG strategy as an alternative approach to creating communicative needs and purposes for learning language. According to CLT, when learners are encouraged to construct their knowledge and control their learning on their own, this will encourage them to no longer process language by simply memorizing the spelling, pronunciation, and meaning of the target words. Instead, they are more likely to use English to communicate their needs and intentions, which may result in more positive learning experiences and thus improved learning performance and motivation (Chang 2005).

### Significance of this study and the proposed strategy—SQG for English language learning

The results of the current study confirmed the effectiveness of the online SQG strategy for enhancing student performance and learning motivation in English. This approach went some way to mitigating the difficulties associated with traditional language teaching methods, especially the communication problems identified by Hwang et al. (2010). In addition, the online SQG strategy was able to meet the call of many scholars to use the target language to create meanings in various contexts (Wang 2009), and encourage the learners to focus on both the meanings and forms of the words they are using (Sanchez 2004). Furthermore, the online SQG strategy meets the criteria and conditions of setting up favorable second language instructional and learning contexts, as identified by Sanchez (2004). These include providing opportunities for learners to be exposed to the language, using it in a communicative context, and being motivated to do so for listening, speaking, writing, and reading. Due to online SQG’s proven potential for second language learning, based on the learning effects obtained in this study, it is suggested that this approach be added to the current CLT strategy reservoir (e.g., along with task-based instruction and output-based production tasks), as an effective communicative approach to learning English as a second language.

### Limitations of this study and suggestions for future works

The present study expands our current knowledge about SQG by confirming its effectiveness with regard to enhancing English performance in relation to the targeted vocabulary items, sentence patterns, and phonics, as well as learning motivation. However, this study investigated the learning effects of sixth-grade students with less than 4 years of formal English learning experience, who worked collaboratively on computers in Taiwan over a period of 11 weeks. The generalizability of the results to other contexts should consequently be exercised with care. Interested researchers are thus advised to extend the current findings by examining the influence of the SQG strategy in second language learning contexts other than English, and involve learners with different language learning experience, at different educational levels, and from different cultures. Moreover, if this strategy is implemented for a longer period of time, it is anticipated that the effects of SQG on all four language areas (i.e., reading, writing, speaking and listening) could be more clearly plotted and understood.
